# An Ecological Survey of Chiggers (Acariformes: Trombiculidae) Associated with Small Mammals in an Epidemic Focus of Scrub Typhus on the China–Myanmar Border in Southwest China

**DOI:** 10.3390/insects15100812

**Published:** 2024-10-16

**Authors:** Ru-Jin Liu, Xian-Guo Guo, Cheng-Fu Zhao, Ya-Fei Zhao, Pei-Ying Peng, Dao-Chao Jin

**Affiliations:** 1 Institute of Pathogens and Vectors, Yunnan Provincial Key Laboratory for Zoonosis Control and Prevention, Dali University, Dali 671000, China; 18187975806@163.com (R.-J.L.); 13330550484@163.com (C.-F.Z.); yf1375315093@163.com (Y.-F.Z.); 2 Institute of Microbiology, Qujing Medical College, Qujing 655100, China; peiyingpeng@hotmail.com; 3 Institute of Entomology, Guizhou University, Guiyang 550025, China; jindaochao@163.com

**Keywords:** chigger mite, vector, ectoparasite, small mammal, ecology, China–Myanmar border

## Abstract

Chiggers (chigger mites) are a group of tiny arthropods, and they are the exclusive vector of *Orientia tsutsugamushi* (Ot), the causative agent of scrub typhus (tsutsugamushi disease). Dehong Prefecture in Yunnan Province of southwest China is located on the China–Myanmar border and is an important focus of scrub typhus. Based on the field surveys in Dehong between 2008 and 2022, the present paper reports the infestation and ecological distribution of chiggers on the body surface of rodents and other sympatric small mammals (shrews, tree shrews, etc.) in the region for the first time. The constituent ratio (*C_r_*), prevalence (*P_M_*), mean abundance (*MA*), and mean intensity (*MI*) were routinely calculated to reflect the infestation of small-mammal hosts with chiggers. Additionally, the species richness (*S*), Shannon–Wiener diversity index (*H*), Simpson dominance index (*D*), and Pielou’s evenness index (*E*) were calculated to illustrate the chigger community structure. Preston’s log-normal model was used to fit the theoretical curve of species abundance distribution, and the Chao 1 formula was used to roughly estimate the expected total species. The “corrplot” package in R software (Version 4.3.1) was used to analyze interspecific relationships, and the online drawing software was used to create a chord diagram to visualize the host–chigger associations. From 1760 small-mammal hosts, a total of 9309 chiggers were identified as belonging to 1 family, 16 genera, and 117 species, with high species diversity. The dominant chigger species were *Leptotrombidium deliense*, *Walchia ewingi*, and *Gahrliepia longipedalis*, with a total *C_r_* = 47.65% (4436/9309), among which *L. deliense* is the most important vector of Ot in China. The overall infestation indexes (*P_M_*, *MA*, and *MI*) and community parameters (*S*, *H*, and *E*) of chiggers in the mountainous areas and outdoors were higher than those in the flatland areas and indoors, with an obvious environmental heterogeneity. *Leptotrombidium deliense* was the dominant species in the flatland and indoors, while *G. longipedalis* was the prevalent species in the mountainous and outdoor areas. The species abundance distribution of the chigger community conformed to log-normal distribution with the theoretical curve equation: ${S(R)}^{\prime }=28e^{{-[0.23(R-0)]}^{2}}$, indicating the existence of many rare species and only a few dominant species in the community. The expected total number of chigger species was roughly estimated to be 147 species, 30 more than the 117 species actually collected, suggesting that some uncommon species may have been missed in the sampling survey. The host–parasite association analysis revealed that one host species can harbor different chigger species, and one chigger species can parasitize different host species with low host specificity. A positive or negative correlation existed among different chigger species, indicating a cooperative or competitive interspecific relationship. The species diversity of chiggers is high in Dehong on the China–Myanmar border, and a large host sample is recommended to find more uncommon species. There is an obvious environmental heterogeneity of the chigger community, with different species diversity and dominant species in different environments. The low host specificity of chiggers and the occurrence of a large number of *L. deliense* in Dehong, especially in flatland areas and indoors, would increase the risk of persistent transmission of scrub typhus in the region.

## Simple Summary

Chiggers are common ectoparasites on rodents and other small mammals, and they transmit scrub typhus, a zoonotic disease. Dehong in Yunnan Province of southwest China is located on the China–Myanmar border, and it is a focus of scrub typhus. The present paper reports the infestation and distribution of chiggers on small mammals in Dehong for the first time. From 1760 rodents and other sympatric small mammals, a total of 9309 chiggers were identified, representing 117 species. Most chigger species had low host specificity. *Leptotrombidium deliense*, a major vector of scrub typhus in China, was the dominant chigger species in Dehong, and it was mainly distributed in flatland areas and indoors. The infestation and community indexes of chiggers in mountainous areas and outdoors were higher than those in flatland areas and indoors. The species abundance distribution of the chigger community conformed to log-normal distribution, and the total number of chigger species was roughly estimated to be 147. The species diversity of the chigger community is high in Dehong, with an obvious environmental heterogeneity. The low host specificity of chiggers and the occurrence of a large number of *L. deliense* in Dehong would increase the transmission risk of scrub typhus on the China–Myanmar border.

## 1. Introduction

Chiggers (chigger mites) are a group of tiny arthropods, and they are the exclusive vector of *Orientia tsutsugamushi* (Akira Tamura et al., 1995), the causative agent of scrub typhus (tsutsugamushi disease) in humans [1,2]. Many chigger species are ectoparasites on the body surface of vertebrates, especially rodents and other small mammals (shrews, tree shrews, etc.) [3,4]. Scrub typhus is a zoonotic disease (zoonosis) caused by *O. tsutsugamushi* (Ot) and transmitted by chiggers [5,6]. Rodents and other sympatric small mammals (e.g., shrews and other insectivores) are the main infectious source and reservoir hosts of Ot. During the food intake of chiggers, Ot can be transmitted among different animal hosts and even from animal hosts to humans [7,8]. Scrub typhus is mainly prevalent in the “tsutsugamushi triangle” regions, including China, which extends from Afghanistan and Pakistan in the west, northern Australia and the southwest Pacific islands in the southeast, to the Korean Peninsula, Japan, and the southeast coast of Russia in the northeast [8,9]. In China, scrub typhus has become a serious public health problem, prevalent in 31 provincial regions of the mainland, and its incidence has been increasing in recent years [10,11]. Dehong Prefecture in Yunnan Province of southwest China is located on the China–Myanmar border, and it is an important focus of scrub typhus in China [10,12]. Myanmar surrounds Dehong in the north, west, and south, and scrub typhus is also prevalent in Myanmar, adjacent to Dehong [13,14,15]. With the frequent economic and trade exchanges between China and Myanmar, the potential risk of scrub typhus spreading from the epidemic foci of Myanmar to Dehong is increasing [16,17]. Besides being the vector of Ot, some chigger species (e.g., *Leptotrombidium scutellare*; Nagayo et al., 1921) are the potential vector of Hantaan virus (HTNV), the pathogen of hemorrhagic fever with renal syndrome (HFRS), a zoonotic disease [2]. Dehong is also a focus of HFRS [18]. Based on the field surveys in Dehong between 2008 and 2022, the present paper reports, for the first time, on the infestation and ecological distribution of chiggers associated with small mammals in the region, being an attempt to provide scientific information for the surveillance and control of vector chiggers and scrub typhus in the border areas.

## 2. Materials and Methods

### 2.1. Field Survey Sites

**Figure 1:**
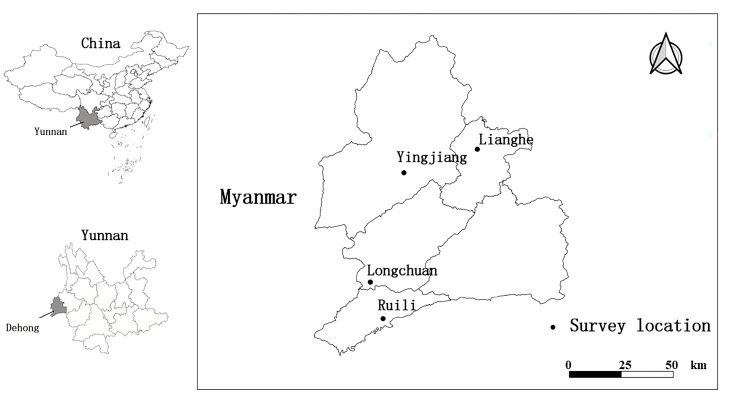
The geographical location and four survey sites of Dehong Prefecture located on the China–Myanmar border in Yunnan Province of southwest China (2008–2022).

Dehong Prefecture (23°50′–25°20′ N, 97°31′–98°43′ E) in Yunnan Province of southwest China is located on the China–Myanmar border, and it is an autonomous administrative region for ethnic minorities Dai and Jingpo [19]. There are five counties in Dehong Prefecture, and the field survey was carried out in four counties, Ruili, Longchuan, Lianghe, and Yingjiang, between 2008 and 2022 (Figure 1).

### 2.2. Chigger Collection and Identification

Mouse traps were placed in different environments to capture rodents (rats, mice, voles, etc.) and other sympatric small mammals (shrews, tree shrews, etc.) in the afternoon or evening. The different environments included the mountainous and flatland areas, the indoors (residential dwelling, livestock barn, poultry shed, etc.), and outdoors (farmland, dry cultivated land, bush area, woodland, etc.). The trapped hosts were collected with white cloth bags the following morning [1,2]. After being conventionally anesthetized, each animal host was separately placed on a large white square plate to collect the chiggers on its body surface. Chiggers are very tiny and difficult to be found by the naked eye, and it is usually challenging to make a complete collection of chiggers from animal hosts. The thin and tender sites of the skin, such as the auricle, outer opening of the external auditory canal, groin, perianal area, and so on, are the familiar places where chiggers often attach. In order to collect as many chiggers as possible and to ensure that the numbers of chiggers collected from each animal host were comparable, the thin and tender skin sites were chosen as the fixed collection sites. Under the help of a magnifier, a lancet or curette (ear scraper) was used to scrape the chiggers and suspected chiggers (chigger-like organisms or some other “dust” and “debris” that look like chiggers) from the skin of each animal host, and the collected chiggers (including suspected chiggers) were preserved in 70% ethanol [20,21]. In the laboratory, the chiggers preserved in 70% ethanol were transferred into distilled water to rinse 2–3 times, and they were then mounted on glass slides with Hoyer’s solution. After dehydration, drying, and transparent processes, each glass slide specimen of the chiggers was carefully observed and measured one-by-one under a microscope (Olympus Corporation, Tokyo, Japan) for species identification [22,23]. The use of animals (including animal euthanasia) for our research was officially approved by the Animals’ Ethics Committee of Dali University, and the representative specimens were deposited in the specimen repository of the Institute of Pathogens and Vectors, Dali University.

### 2.3. Statistics of Chigger Infestation and Community Indexes

The constituent ratio (*C_r_*) was conventionally used to calculate the percentage of a certain chigger or host species in the community. The prevalence (*P_M_*) was used to calculate the infestation frequency of small-mammal hosts with chiggers, the percentage of infested hosts. The mean abundance (*MA*) was used to calculate the average infestation intensity of chiggers on the examined hosts (chiggers per examined host), and *MI* was used to calculate the average infestation intensity of chiggers on the infested hosts (chiggers per infested host). The Chi-square test was used to compare the prevalence (*P_M_*), and the non-parametric Kruskal–Wallis test to analyze the mean abundance (*MA*) and mean intensity (*MI*). When *p* < 0.05, it was considered statistically significant; otherwise, it was not [7,23]. Four commonly used community parameters were calculated to reflect the community structure of chiggers, in which the species richness index (*S*) stands for the number of species within a community, Shannon–Wiener’s diversity index (*H*′) and Pielou’s evenness (*E*) represent the diversity and distribution evenness of species within the community, and Simpson’s dominance index (*D*) reflects the predominant position of dominant species within the community [24,25].

(1) $$C_{r}=\frac{N_{i}}{N}\times 100\%$$

(2) $$P_{M}=\frac{H_{i}}{H}\times 100\%$$

(3) $$MA=\frac{N_{i}}{H}$$

(4) $$MI=\frac{N_{i}}{H_{i}}$$

(5) $$H^{\prime }=-\sum_{i=1}^{S}(\frac{N_{i}}{N})\ln\frac{N_{i}}{N}$$

(6) $$E=\frac{H^{\prime }}{\ln S}$$

(7) $$D=\sum_{i=1}^{S}{\left(\frac{N_{i}}{N}\right)}^{2}$$

In the above formulas, *S* = the number of species in the community, *N_i_* = the number of a certain species (species *i*), *N* = the total number of all the species, *H_i_* = the number of hosts infested with chiggers, and *H* = the total number of hosts examined.

### 2.4. Theoretical Curve Fitting of Species Abundance Distribution of Chigger Community

The species abundance distribution illustrates the relationship between the number of species and individuals in a particular community [25,26]. In the present study, all the chiggers in Dehong Prefecture were regarded as a chigger community unit. In a semi-logarithmic coordinate system, the X-axis with logarithmic scales based on log3N was used to mark the number of chigger individuals, and the Y-axis with arithmetic scales was used to mark the number of chigger species. Preston’s log-normal distribution model was used to fit the theoretical curve of species abundance distribution of the chigger community using the following formulas [22,27]:

(8) $$S{\left(R\right)}^{\prime }=S_{0}e^{{-[\alpha (R-R_{0})]}^{2}}$$

(9) $$R^{2}=1-\frac{\sum_{R=0}^{m}{\left[S\left(R\right)-SR^{\prime }\right]}^{2}}{\sum_{R=0}^{m}{\left[S\left(R\right)-SR^{\prime \prime }\right]}^{2}}$$

(10) $$S{\left(R\right)}^{\prime \prime }=\frac{1}{m}\sum_{R=0}^{m}S(R)$$

In the above formulas, *S*(*R*)′ = the theoretical number of chigger species at the *R*-th log interval, *S*_0_ = the number of chigger species at the mode log interval (*R*_0_), α = the distribution expansion constant, which is determined according to the best goodness (*R*^2^) of the curve fitting, *S*(*R*) = the actual number of chigger species at the *R*-th log interval, and *S*(*R*)′′ = the average number of chigger species for each log interval.

### 2.5. Estimation Method of Total Species Based on Rare Species

The Chao 1 method based on rare species was used to roughly estimate the expected total number (theoretical total number) of chigger species [20,28]:

(11) $$S^{*}=S_{obs}+\frac{\alpha^{2}}{2b}$$

In the above formula, S* = the expected total number of chigger species, *S_obs_* = the number of chigger species actually collected in the field survey, *a* = the number of rare species with only one individual collected, and *b* = the number of rare species with only two individuals collected.

### 2.6. Host–Chigger and Chigger–Chigger Relationships

The data of chigger species and their corresponding host species were input into the Flourish online mapping software platform (https://flourish.studio (accessed on 14 December 2023)). Additionally, a chord diagram was created to visualize the host–parasite association (host–chigger relationship) between different host species and chigger species. Based on Spearman’s correlation coefficient (*r*), the “corrplot” statistical package in R software (Version 4.3.1) was used to visualize the interspecific relationships between different chigger species, the chigger–chigger relationships [29].

## 3. Results

### 3.1. Species Composition of Small-Mammal Hosts

Between 2008 and 2022, 1760 small-mammal hosts were captured from the 4 survey sites in Dehong Prefecture. They were identified as belonging to 9 families, 16 genera, and 27 species included in the orders Rodentia, Eulipotyphyla, and Scandentia. Rodents were the majority of small-mammal hosts. The number of rodent species accounted for 70.37% of the total host species (the constituent ratio *C_r_* = 70.37%, 19/27), and the individual rodents accounted for 84.43% of the total host individuals (*C_r_* = 84.43%, 1486/1760; Table 1). Of the 27 host species identified, *Rattus tanezumi* (Temminck, 1845) (*C_r_* = 52.10%, 917/1760) and *Eothenomys miletus* (Thomas, 1914) (*C_r_* = 16.82%, 296/1760) were two dominant species, and *Suncus murinus* (Linnaeus, 1766) (*C_r_* = 7.16%, 126/1760) and *Rattus andamanensis* (Blyth, 1860) (*C_r_* = 6.93%, 122/1760) came next. The rest 23 host species included 16 rodent species, six insectivore species and one scandent species. The 16 rodent species are *Rattus nitidus* (Hodgson, 1845); *R. norvegicus* (Berkenhout, 1769); *Mus caroli* Bonhote, 1902; *M. musculus* Linnaeus, 1758; *M. pahari* Thomas, 1916; *Niviventer andersoni* (Thomas, 1911); *N. confucianus* (Hodgson, 1871); *N. fulvescens* (Gray, 1847); *Apodemus chevrieri* Milne-Edwards, 1868; *Bandicota indica* (Bechstein, 1800); *Hylopetes alboniger* (Hodgson, 1836); *Dremomys pernyi* (Milne-Edwards, 1867); *E. olitor* (Thomas, 1911); *Berylmys manipulus* (Thomas, 1916); *Rhizomys sinensis* Gray, 1831; and *R*. *pruinosus* Blyth, 1851. The six insectivore species are *Crocidura dracula* Thomas, 1912; *C. attenuata* Milne-Edwards, 1872; *C. tadae* Allen, 1923; *Anourosorex squamipes* Milne-Edwards, 1872; *Hylomys suillus* Mueller, 1840; and *Parascaptor leucura* (Blyth, 1850) in Eulipotyphyla. The scandent species is *Tupaia belangeri* (Wagner, 1841). 

### 3.2. Species Composition of Chiggers

**Figure 2:**
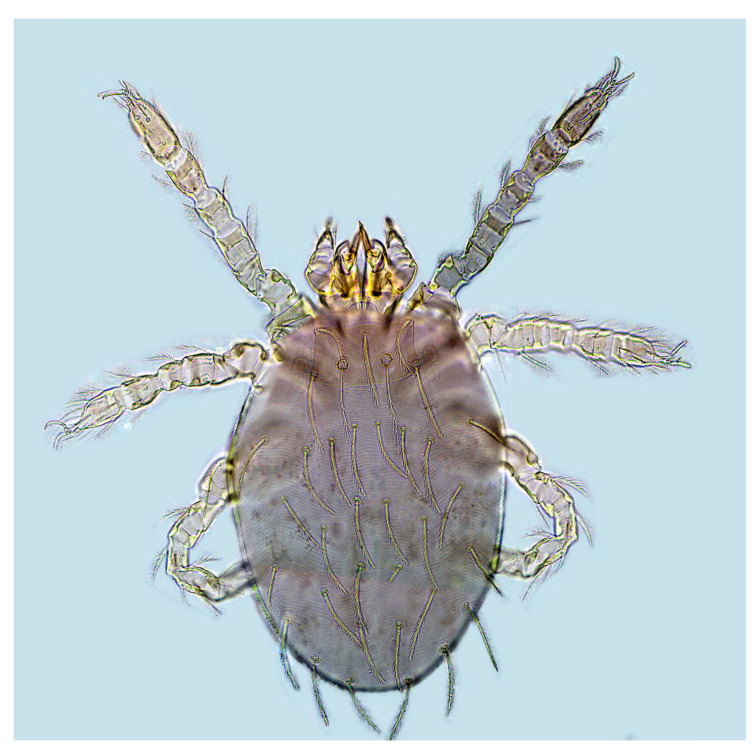
A photo of *L*. *deliense* (×1000), one of the three dominant chigger species in Dehong Prefecture on the China–Myanmar border in Yunnan Province of southwest China (2008–2022).

**Figure 3:**
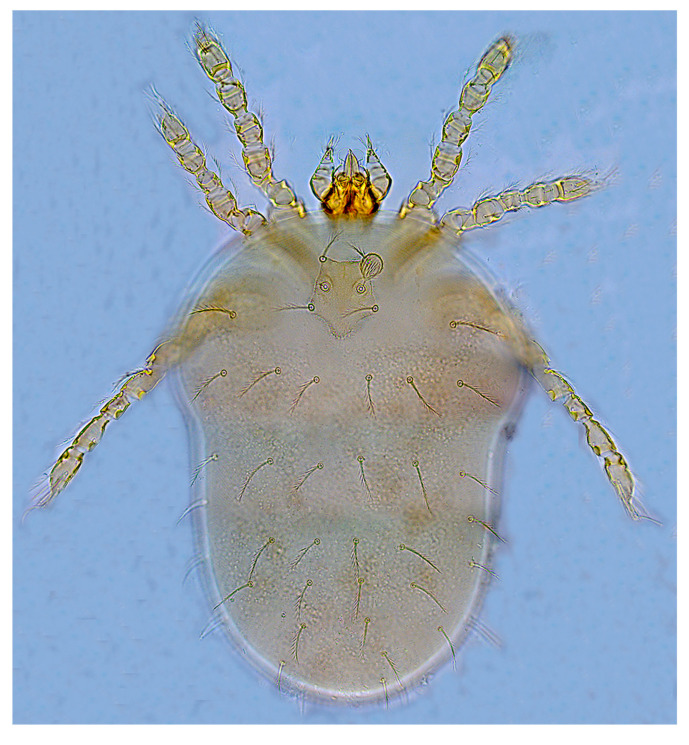
A photo of *W. ewingi* (×1000), one of the three dominant chigger species in Dehong Prefecture on the China–Myanmar border in Yunnan Province of southwest China (2008–2022).

**Figure 4:**
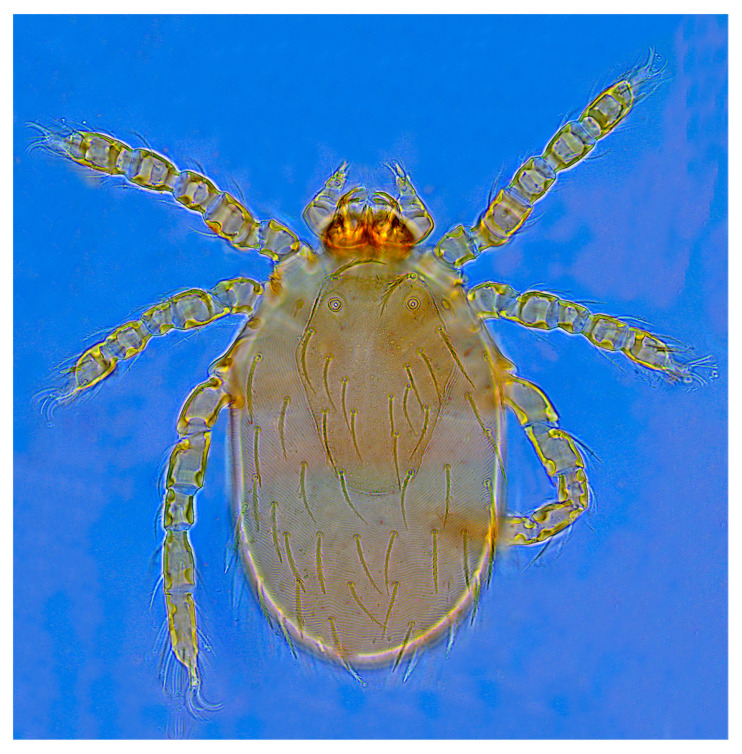
A photo of *G. longipedalis* (×1000), one of the three dominant chigger species in Dehong Prefecture on the China–Myanmar border in Yunnan Province of southwest China (2008–2022).

A total of 9309 collected chiggers were identified as belonging to 16 genera and 117 species in the family Trombiculidae. The genus *Leptotrombidium* had the highest number of species (44 species) and individuals (3167 mites), followed by *Gahrliepia* and *Walchia* (Table 2). Of the 117 chigger species identified, *Leptotrombidium deliense* Walch, 1922 (*C_r_* = 17.16%, 1597/9309), *Walchia ewingi* Fuller, 1949 (*C_r_* = 16.22%, 1510/9309) and *Gahrliepia longipedalis* Yu et Yang, 1986 (*C_r_* = 14.28%, 1329/9309) were the three dominant species, with the total constituent ratio *C_r_* = 47.65% (4436/9309). The diagnostic characteristics and photos of these dominant chigger species are shown in Table 3 and Figure 2, Figure 3 and Figure 4. Of the 117 chigger species identified, 12 species are the vectors or potential vectors of scrub typhus, and these vector species are *L. deliense*; *L. scutellare*; *L. rubellum* Wang et Liao, 1984; *L. sialkotense* Vercammen-Grandjean and Langston, 1976 (*L. jishoum* Wen, Li, Zhang and Liao, 1988); *L. imphalum* Vercammen-Grandjean et Langston, 1975; *L. rusticum* Yu, Yang et Gong, 1986; *L. fuji* Kuwata et al, 1950; *L. apodemi* Wen et Sun, 1984; *L. intermedium* Nagayo et al, 1920; *Helenicula simena* (Hsu et Chen, 1957); *Ascoschoengastia indica* Hirst, 1915; and *Schoengastiella ligula* Radford, 1946. Of the 12 vector species, *L. deliense*, *L. scutellare*, *L. rubellum* and *L. sialkotense* are the four of six main vectors of scrub typhus in China. The distribution of these vector species was uneven at different survey sites, and the numbers of the vector species in Ruili (1341) and Longchuan (1021) were much more than those at the rest two sites (Yingjiang and Lianghe). Among the 12 vector species, *L. deliense* had the highest constituent ratio (*C_r_* = 61.88%), and *L. rusticum* and *H. simena* came next (Table 4). 

### 3.3. Chigger Infestation and Community on Four Main Host Species

The overall indexes and community parameters of chiggers varied on the four main host species: *R. tanezumi*, *E. miletus*, *S. murinus*, and *R. andamanensis*. The indexes of infestation and diversity of chiggers on *E. miletus* (*P_M_* = 46.96, *MA* = 14.25, *MI* = 30.34, and *H*’ = 2.71) were much higher than those on *R. tanezumi*, *S. murinus*, and *R. andamanensis*. The number of chigger species was the highest on *R. tanezumi* (*S* = 75), much higher than that of the other three host species (Table 5).

The infestation indexes of the three dominant chigger species (*L. deliense*, *W. ewingi*, and *G. longipedalis*) on the four main host species were also very different. *Leptotrombidium deliense* was found on all four main host species, and it was one of the dominant chigger species on *R. tanezumi* and *S. murinus* (Table 6).

### 3.4. Chigger Infestation and Community in Different Environments

The overall infestation indexes and community parameters of chiggers varied in different environments. The species and individuals of chiggers were higher in the mountain areas (95 species and 7425 individuals) than in the flatland areas (61 species and 1884 individuals). The overall infestation indexes of chiggers (*P_M_*, *MA*, and *MI*) were higher in the mountainous areas than in the flatland areas (*p* < 0.05; Table 7). The species richness (*S* = 95), Shannon–Weiner’s diversity index (*H*’ = 2.95), and Pielou’s evenness index (*E* = 0.65) of the chigger community were also higher in the mountainous areas than in the flatland areas (*S* = 61, *H*’ = 2.07, and *E* = 0.50; Table 9). The species and individuals of chiggers were higher in the outdoors (98 species and 7620 individuals) than in the indoors (68 species and 1689 individuals). The overall infestation indexes (*P_M_*, *MA*, and *MI*) and community parameters (*S*, *H*’, and *E*) of chiggers were also higher in the outdoors than in the indoors (*p* < 0.05; Table 8 and Table 9). *Gahrliepia longipedalis*, *W. ewingi*, and *L. deliense* were the three dominant chigger species in the mountainous areas and outdoors. *Leptotrombidium deliense*, *W. ewingi*, and *S. ligula* (Radford, 1946) were the dominant chigger species in the flatland areas and indoors (Table 10 and Table 11).

### 3.5. Species Abundance Distribution of Chigger Community

**Figure 5:**
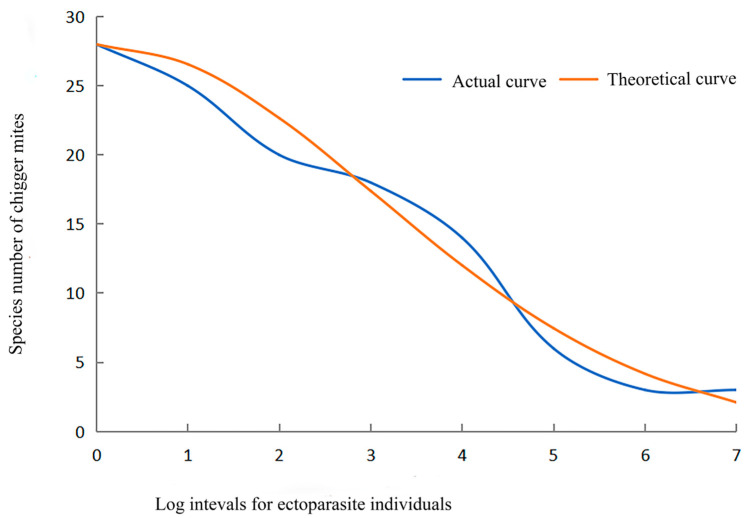
Theoretical curve fitting of species abundance distribution of the chigger community in Dehong prefecture on the China–Myanmar border in Yunnan Province of southwest China (2008–2022).

The species abundance distribution of the chigger community was successfully fitted with Preston’s log-normal model, with the fitting goodness *R*^2^ = 0.99. The theoretical curve equation was: ${S(R)}^{\prime }=28e^{{-[0.23(R-0)]}^{2}}$. The result indicated that the species abundance distribution of the chigger community conformed to log-normal distribution, and many rare species existed in the community, with only a few dominant species. The theoretical curve of the species abundance revealed that the number of chigger species gradually decreased with the increase in chigger individuals (Table 12 and Figure 5).

### 3.6. Expected Total Number of Chigger Species

Of the 117 chigger species identified, 28 species had only 1 individual, and 13 species had 2 individuals. According to the Chao 1 estimation method, the expected total number of chigger species in Dehong Prefecture was roughly estimated to be 147, 30 more than the actual identified 117 species.

### 3.7. Chigger–Chigger Relationships

**Figure 6:**
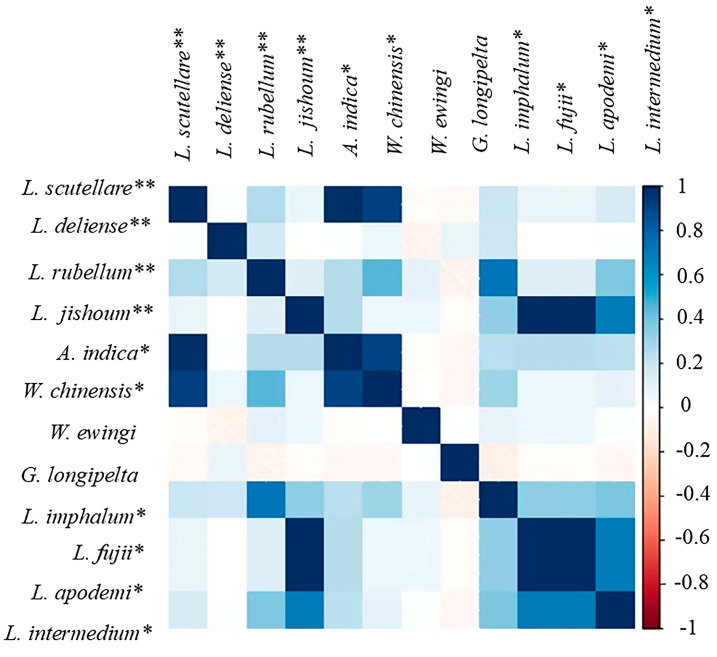
Interspecific relationships of chiggers on small mammals in Dehong Prefecture on the China–Myanmar border in Yunnan Province of southwest China (2008–2022). Annotation: The chigger species marked with “**” are the main vectors of *O*. *tsutsugamushi* (Ot), the causative agent of scrub typhus (tsutsugamushi disease) in China, and those with “*” are the potential vectors of Ot.

The “corrplot” package in R software (Version 4.3.1) was used to analyze and visualize the interspecific relationships among 12 important chigger species (dominant species or vector species) on small mammals. The confidence interval was 0.95. As shown in Figure 6, the blue squares represent the positive correlations between any two chigger species, and the pink ones indicate the negative correlations. The color depth indicates the degree of the positive or negative correlation. The values for the positive correlation ranged from 0 to 1 (0, 1), and the values for the negative correlation were from 0 to −1 (0, −1; Figure 6). *Leptotrombidium deliense*, one of the dominant chigger species in the present study and the most important vector of Ot in China, showed a slightly positive correlation with *L. imphalum* and *L. rubellum* (two of the six main vectors of Ot in China). *Leptotrombidium scutellare*, also a very important vector (second only to *L. deliense*) of Ot in China, showed an obvious positive correlation with *A. indica* and *W. chinensis* (Figure 6).

### 3.8. Host–Chigger Relationships

**Figure 7:**
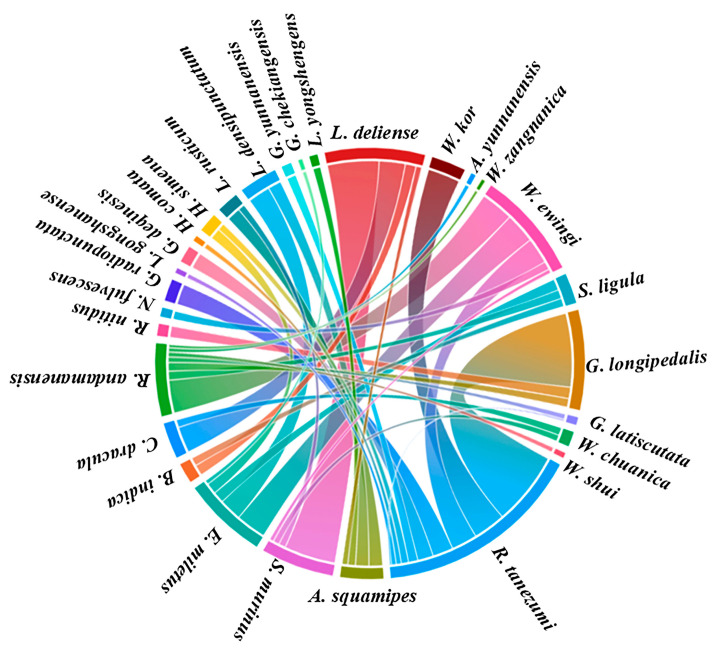
The chord diagram of host–chigger relationships in Dehong Prefecture on the China–Myanmar border in Yunnan Province of southwest China (2008–2022).

By using Flourish online mapping software, a chord diagram was created to visualize the host–parasite association between the 9 main small-mammal hosts and 20 main chigger species (Figure 7). The nine main host species were *R. tanezumi*, *A. squamipes*, *S. murinus*, *E. miletus*, *B. indica*, *C. dracula*, *R. andamanensis*, *R. nitidus*, and *N. fulvescens.* The 20 main chigger species are *Gahrliepia radiopunctata* Hsu et al., 1965; *Leptotrombidium gongshanense* Yu et al., 1981; *Gahrliepia deqinensis* Yu et Yang, 1982; *H. comata*; *H. simena*; *L. rusticum*; *Leptotrombidium densipunctatum* Yu et al., 1982; *Gahrliepia yunnanensis* Hsu et al., 1965; *Gahrliepia chekiangensis* Chu, 1964; *Leptotrombidium yongshengense* Yu et Yang, 1986; *L. deliense*, *Walchia kor* (Chen et Hsu, 1957); *Ascoschoengastia yunnanensis* Yu et al., 1980; *Walchia zangnanica* Wu et Wen, 1984; *W. ewingi*; *S. ligula*; *G. longipedalis*; *Gahrliepia latiscutata* Chen et Fan, 1981; *Walchia Chuanica* Wen et Song, 1984; and *Walchia shui* Wen et Song, 1984. In Figure 7, the color ribbons and strings represent the host–parasite associations (host–chigger relationships) between different chigger species and their corresponding host species. The width and thickness of the ribbons and strings represent the number of chiggers and hosts. The results showed that a certain chigger species could select various small mammal species as its hosts, and a specific host species could harbor a number of chigger species (Figure 7).

## 4. Discussion

Chiggers are the exclusive vector of Ot, the pathogen of scrub typhus, and they can also serve as the potential vector of HV, the pathogen of HFRS. Dehong Prefecture in the present study is an important focus of scrub typhus and HFRS. Being one of the 16 administrative regions of Yunnan Province in southwest China, Dehong Prefecture is located on the China–Myanmar border [12,30,31]. There are five counties in Dehong Prefecture, and the present study surveyed four out of the five counties. Of the four counties surveyed, Ruili, Longchuan, and Yingjiang are the three counties directly bordering Myanmar, being busy trade and tourism areas with frequent human flow [32,33]. Ruili and Longchuan are also important surveillance areas for infectious diseases (including zoonotic diseases) in the border regions [16,17,30]. In the present survey, the majority of vector chigger species mainly came from Ruili and Longchuan (Table 4), implying a high potential risk of transmitting scrub typhus in the two counties. Scrub typhus is prevalent in northern Myanmar bordering Dehong, and the disease can be imported from northern Myanmar to Dehong at any time [13,14]. The present study described an ecological survey of chiggers on small mammals in Dehong for the first time, and it will benefit the surveillance and control of scrub typhus and vector chiggers in the border areas.

Dehong is a prefecture under the jurisdiction of Yunnan Province, China. The 117 chigger species identified from Dehong greatly exceed the number of chigger species recorded in some other provinces of China, e.g., 41 species in Hubei Province [34], 17 species in Shandong Province [35], and 81 species in northwest China, which covers 5 provincial regions (Shanxi, Ningxia, Gansu, Qinghai, and Xinjiang) [36]. The result suggests that the species diversity of chiggers is very high in Dehong, which may be associated with the geographical location and climate in the region. Dehong Prefecture is located at the south end of the Gaoligong Mountains, with a complex topography and ecological environment and high species diversity of small mammals [37,38,39], and this may be an important factor leading to the high species diversity of chiggers in Dehong. The majority of Dehong’s territory belongs to tropical and subtropical regions. The warm and humid climate in Dehong is beneficial to the growth, development, and reproduction of many chigger species [19,40,41].

Being the four main species of small-mammal hosts in Dehong Prefecture, *R. tanezumi*, *E. miletus*, *S. murinus*, and *R. andamanensis* are important infectious sources and reservoir hosts of many zoonoses, including scrub typhus and HFRS [40,41,42,43]. Of the three dominant chigger species (*L. deliense*, *W. ewingi*, and *G. longipedalis*) in Dehong, *L. deliense* was the most dominant one, with the highest constituent ratio (*C_r_* = 17.16%, 1597/9309), and it is not only a major vector of scrub typhus in China, but also an important vector of the disease in many parts of the world [40,41,42]. Among the 12 vector species found in Dehong, *L. deliense* also had the highest constituent ratio (Table 4). The co-existence of the 12 vector species with a large number of *L. deliense* in Dehong would increase the persistent preservation of Ot among small-mammal hosts and the potential transmission risk of Ot from small mammals to humans through the vector chiggers.

As shown in Table 10 and Table 11, the constituent ratios of *L. deliense* were much higher in the flatland areas and indoors than in the mountainous areas and outdoors. *Schoengastiella ligula* was mainly found in the flatland areas and indoors, and it is suspected to be a potential vector of Ot [40,44]. The environment of indoors is closely related to humans’ daily life. In Dehong Prefecture and other parts of Yunnan Province in southwest China, the majority of cultivated farmlands are distributed in flatland areas [45,46]. Humans have a lot of opportunity to be invaded by chiggers on rodents and other sympatric small mammals in their farming activities. The results of the present study imply that the potential transmission risk of Ot would be much higher in the flatland areas and indoors than in the mountainous areas and outdoors in Dehong Prefecture. We should pay more attention to the flatland areas and indoors in the surveillance and control of scrub typhus in Dehong Prefecture.

The species abundance distribution of a community describes the relationship between the number of species and the number of individuals in a specific community [22,26]. Preston’s log-normal distribution model is often used to fit the species abundance distribution theoretical curve [24,27]. In the present study, the species abundance distribution of the chigger community was successfully fitted with Preston’s log-normal model, with a very high fitting goodness (*R*^2^ = 0.99), which indicates that the chigger community in Dehong Prefecture conformed to a log-normal distribution pattern. The theoretical curve of the species abundance distribution showed that the number of chigger species gradually decreased with the increase in chigger individuals (Table 12 and Figure 5). The result indicates that most chigger species in Dehong were rare or uncommon species with few (even one or two) individuals, and only a few species were dominant ones with abundant individuals.

There are several methods for predicting the expected total number of species in a community, and the Chao 1 method used in the present study is one of them. In comparison with some other methods, the Chao 1 formula is a simple and well-proven one, and it has been widely used in ecological research [20,28]. Based on the Chao 1 method, the expected total number of chigger species in Dehong Prefecture was roughly estimated to be 147, 30 more than the 117 species actually identified. The result suggests that some uncommon species may have been potentially missed (not found) in the sampling survey [24,47]. To find more uncommon species, a large host sample is recommended. In ecological practice, it is very difficult to exactly estimate the total number of species within a community, no matter what formula is used. The estimate in the present study is only a rough figure and is unlikely to be an accurate result [48,49].

In the present study, the “corrplot” package in R software (Version 4.3.1) was used to analyze and visualize the interspecific relationship between any two different chigger species, the chigger–chigger relationship. The result showed that positive or negative correlations existed among different chigger species in the selection of small-mammal hosts, indicating that the chigger species with positive correlations have a tendency to co-exist on the same hosts, and the chigger species with negative correlations tend to choose different hosts [29].

In the present study, Flourish online mapping software was used to create a chord diagram to visualize the host–parasite association (host–chigger relationship) between small-mammal hosts and chiggers. The result showed that a certain chigger species could select different small-mammal species as its hosts at the same time, and a specific host species could harbor a number of chigger species (Figure 7), indicating the low host specificity of chiggers [2,23].

## Figures and Tables

**Table 1:** Identification of small-mammal hosts in Dehong Prefecture on the China–Myanmar border in Yunnan Province of southwest China (2008–2022).

Orders of Small-Mammal Hosts	Families and Genera of Small-Mammal Hosts		Species and Constituent Ratios (*C_r_*, %) of Small-Mammal Hosts		Individuals and Constituent Ratios (*C_r_*, %) of Small-Mammal Hosts	
	No. of Families	No. of Genera	No. of Species	*C_r_*, %	Individuals	*C_r_*, %
Rodentia	5	10	19	70.37	1486	84.43
Eulipotyphla	3	5	7	25.93	273	15.51
Scandentia	1	1	1	3.70	1	0.06
Total	9	16	27	100.00	1760	100.00

**Table 2:** Taxonomic identification of chiggers from small mammals in Dehong Prefecture on the China–Myanmar border in Yunnan Province of southwest China (2008–2022).

Genera of Chiggers	Number of Chigger Species and Constituent Ratios (*C_r_*, %)		Chigger Individuals and Constituent Ratios (*C_r_*, %)	
	No. of Species	*C_r_*, %	Individuals	*C_r_*, %
*Leptotrombidium*	44	37.61	3167	34.02
*Trombiculindus*	2	1.71	59	0.64
*Neotrombicula*	1	0.85	1	0.01
*Chiroptella*	1	0.85	7	0.08
*Lorillatum*	2	1.71	40	0.43
*Helenicula*	12	10.26	481	5.17
*Paradoloisia*	1	0.85	1	0.01
*Ascoschoengastia*	5	4.28	44	0.47
*Walchiella*	1	0.85	45	0.48
*Mackiena*	1	0.85	1	0.01
*Herpetacarus*	5	4.28	30	0.32
*Schoengastia*	2	1.71	2	0.02
*Subtotal*	77	65.81	3878	41.66
*Walchia*	15	12.83	2607	28.00
*Schoengastiella*	3	2.56	253	2.72
*Gahrliepia*	19	16.24	2508	26.94
*Intermedialia*	3	2.56	63	0.68
*Total*	117	100.00	9309	100.00

**Table 3:** The diagnostic characteristics of three dominant chigger species in Dehong Prefecture on the China–Myanmar border in Yunnan Province of southwest China (2008–2022).

Names of Dominant Chigger Species	Diagnostic Characteristics
*L. deliense*	fPp = N/N/BNN; Pc = 3; Gn = 2; fSc: PL > AM > AL; SB/PL; fCx = 1.1.1; fSt = 2.2; fD = 2H-8-6-6-4-2; DS = 28; VS = 20–22; NDV = 48–50; Ip = 626–719; AW 55–66, PW 64–78, SB 26–31, ASB 24–30, PSB 12–15, SD 37–43, AP 24–30, AM 44–59, AL 34–46, PL 44–58, S 62–80, H 46–58, Dmin 34–46, Dmax 42–58, Vmin 27–32, Vmax 39–52, pa 220–255, pm 186–215, pp 215–255.
*W. ewingi*	fPp = N/N/NNN; Gn = 2; fSc: PL > AL; SB/PL; fCx = 1.1.3; DS = 36–42; VS = 47–60; NDV = 83–102; Ip = 494–551; AW 26–34, PW 36–45, SB 18–26, ASB 20–23, PSB 33–36, SD 53–59, AP 30–36, AL 23–29, PL 25–31, S 23–27 × 14–15, pa 178–193, pm 142–160, pp 174–198.
*G. longipedalis*	fPp = B/B/NNN; Pc = 3; Gn = 2; fSc: PL > AL; SB/PL; fCx = 7.6.6; fD = 2H-6-10-8-6-6-6-4-2; DS = 45–50; VS = 63–69; NDV = 108–119; Ip = 983–1021; AW 63–64, PW 111–114, SB 62–67, ASB 30–31, PSB 168–178, SD 198–209, AP 51–54, AL 52–57, PL 63–69, S 44–51 × 10–13, pa 330–333, pm 288–310, pp 365–378.

**Table 4:** Numbers (No.) and constituent ratios (*C_r_*) of vector chigger species at different survey sites on the China–Myanmar border in Yunnan Province of southwest China (2008–2022).

Species of Vector Chiggers	No. of Chiggers at Different Survey Sites				Total No. and *C_r_* of Chiggers	
	Ruili	Longchuan	Yingjiang	Lianghe	No.	*C_r_*, %
*L. deliense* **	672	833	0	92	1597	61.88
*L. scutellare* **	17	1	0	0	18	0.70
*L. rubellum* **	15	6	0	0	21	0.81
*L. jishoum* **	0	0	1	0	1	0.04
*L. imphalum* *	20	9	0	0	29	1.12
*L. rusticum* *	234	7	0	102	343	13.29
*L. fuji* *	0	1	0	0	1	0.04
*L. apodemi* *	0	1	0	0	1	0.04
*L. intermedium* *	0	1	0	1	2	0.08
*H. simena* *	135	156	0	14	305	11.82
*A. indica* *	14	3	1	0	18	0.70
*S. ligula*	234	3	0	8	245	9.94
Total	1341	1021	2	217	2581	100.00

Annotation: Chigger species marked with “**” are the main vectors of scrub typhus in China, and those with “*” are the potential vectors of the disease.

**Table 5:** Overall infestation indexes and community parameters of chiggers on four main host species on the China–Myanmar border in Yunnan Province of southwest China (2008–2022).

Names of Main Host Species	No. of Hosts	Overall Infestation Indexes of Chiggers			Community Parameters of Chiggers			
		*P_M_*	*MA*	*MI*	*S*	*H*′	*E*	*D*
*R. tanezumi*	917	17.56	2.50	14.25	75	2.38	0.55	0.18
*E. miletus*	296	46.96	14.25	30.34	66	2.71	0.65	0.12
*S. murinus*	126	6.35	0.63	10.00	12	1.42	0.57	0.44
*R. andamanensis*	122	38.52	9.90	25.70	43	2.22	0.22	0.22

**Table 6:** Infestation indexes of the three dominant chigger species on four main host species in Dehong Prefecture on the China–Myanmar border in Yunnan Province of southwest China (2008–2022).

Four Main Host Species	Three Dominant Chigger Species	No. of Chiggers	*C_r_*, %	*P_M_*, %	*MA*	*MI*
*R. tanezumi* (*n* = 917)	*L. deliense*	809	35.27	4.36	0.88	20.23
	*W. ewingi*	476	20.75	3.60	0.52	14.42
	*G. longipedalis*	17	0.74	0.76	0.02	2.43
*E. miletus* (*n* = 296)	*L. deliense*	91	2.16	4.73	0.31	6.50
	*W. ewingi*	406	9.63	16.89	1.37	8.12
	*G. longipedalis*	1153	27.34	20.95	3.90	18.60
*S. murinus* (*n* = 126)	*L. deliense*	52	65.00	3.17	0.41	13
	*W. ewingi*	0	-	-	-	-
	*G. longipedalis*	6	7.50	1.59	0.05	3
*R. andamanensis* (*n* = 122)	*L. deliense*	169	14.04	12.30	1.39	11.27
	*W. ewingi*	513	42.61	25.41	4.21	16.55
	*G. longipedalis*	35	2.91	11.48	0.29	2.5

**Table 7:** Overall infestation indexes of chiggers on small mammals in the flatland and mountainous areas of Dehong Prefecture on the China–Myanmar border in Yunnan Province of southwest China (2008–2022).

The Flatland and Mountainous Areas	Small-Mammal Hosts		Chiggers		Overall Infestation Indexes of Chiggers		
	Examined Individuals	Infested Individuals	No. of Species	Individuals	*P_M_*	*MA*	*MI*
Flatland areas	965	126	61	1884	13.06	1.95	14.95
Mountainous areas	795	324	95	7425	40.75	9.34	22.92

**Table 8:** Overall infestation indexes of chiggers on small mammals in the indoors and outdoors of Dehong Prefecture on the China–Myanmar border in Yunnan Province of southwest China (2008–2022).

The Indoors and Outdoors	Small-Mammal Hosts		Chiggers		Overall Infestation Indexes of Chiggers		
	Examined Individuals	Infested Individuals	No. of Species	Individuals	*P_M_*	*MA*	*MI*
Indoors	1032	139	68	1689	13.47	1.64	12.15
Outdoors	728	311	98	7620	42.72	10.47	24.50

**Table 9:** Community parameters of chiggers on small mammals in different environments of Dehong Prefecture on the China–Myanmar border in Yunnan Province of southwest China (2008–2022).

Different Environments	Community Parameters of Chiggers			
	*S*	*H’*	*D*	*E*
Flatland areas	61	2.07	0.22	0.50
Mountainous areas	95	2.95	0.09	0.65
Indoors	68	2.19	0.22	0.52
Outdoors	98	2.97	0.09	0.65

**Table 10:** Overall infestation indexes of dominant chigger species in the flatland and mountainous areas of Dehong Prefecture on the China–Myanmar border in Yunnan Province of southwest China (2008–2022).

The Flatland and Mountainous Areas	Dominant Chigger Species	No. of Chiggers	*C_r_*, %	*P_M_*	*MA*	*MI*
Flatland areas	*L*. *deliense*	668	34.46	3.52	0.69	19.65
	*W*. *ewingi*	504	26.75	2.69	0.52	19.34
	*S. ligula*	236	12.53	4.66	0.24	5.24
Mountainous areas	*G. longipedalis*	1309	17.63	7.42	1.65	13.09
	*W. ewingi*	1006	13.55	13.21	1.27	9.58
	*L. deliense*	929	12.51	12.58	1.17	15.75

**Table 11:** Overall infestation indexes of dominant chigger species in the indoors and outdoors of Dehong Prefecture on the China–Myanmar border in Yunnan Province of southwest China (2008–2022).

The Indoors and Outdoors	Dominant Chigger Species	No. of Chiggers	*C_r_*, %	*P_M_*	*MA*	*MI*
Indoors	*L. deliense*	574	33.98	5.22	0.79	15.11
	*W. ewingi*	511	30.25	3.71	0.70	18.93
	*S. ligula*	137	8.11	4.95	0.19	3.81
Outdoors	*G. longipedalis*	1310	17.19	9.79	1.27	12.97
	*L. deliense*	1023	13.43	5.33	0.99	18.60
	*W. ewingi*	999	13.11	10.08	0.97	9.61

**Table 12:** The fitting results of species abundance distribution of the chigger community on small mammals in Dehong prefecture on the China–Myanmar border in Yunnan Province of southwest China (2008–2022).

Log Intervals Based on log_3_*N*	Individual Ranges of Chiggers at Each Log Interval	Midpoint Values of Chigger Individuals at Each Log Interval	Actual Chigger Species	Theoretical Chigger Species
0	0–1	1	28	28.00
1	2–4	3	25	26.56
2	5–13	9	20	22.66
3	14–40	27	18	17.39
4	41–121	81	14	12.01
5	122–364	243	6	7.46
6	365–1093	729	3	4.17
7	1094–3280	2187	3	2.10

## Data Availability

The experimental data used to support the findings of this study are available from the corresponding author upon request.

## References

[1] Lv Y., Guo X.G., Jin D.C., Song W.Y., Peng P.Y., Lin H., Fan R., Zhao C.F., Zhang Z.W., Mao K.Y. (2021). Infestation and seasonal fluctuation of chigger mites on the Southeast Asian house rat (*Rattus brunneusculus*) in southern Yunnan Province, China. Int. J. Parasitol. Parasites Wildl..

[2] Song W.Y., Lv Y., Yin P.W., Yang Y.Y., Guo X.G. (2023). Potential distribution of *Leptotrombidium scutellare* in Yunnan and Sichuan Provinces, China, and its association with mite-borne disease transmission. Parasites Vectors.

[3] Chen K.Y., Roe R.M., Ponnusamy L. (2022). Biology, systematics, microbiome, pathogen transmission and control of chiggers (Acari: Trombiculidae, Leeuwenhoekiidae) with emphasis on the United States. Int. J. Environ. Res. Public Health.

[4] Li B., Guo X.G., Zhao C.F., Zhang Z.W., Fan R., Peng P.Y., Song W.Y., Ren T.G., Zhang L., Qian T.J. (2022). Infestation of chigger mites on Chinese mole shrew, *Anourosorex squamipes*, in Southwest China and ecological analysis. Parasite.

[5] Weitzel T., Aylwin M., Martínez-Valdebenito C., Jiang J., Munita J.M., Thompson L., Abarca K., Richards A.L. (2018). Imported scrub typhus: First case in South America and review of the literature. Trop. Dis. Travel Med. Vaccines.

[6] Paris D.H., Shelite T.R., Day N.P., Walker D.H. (2013). Unresolved problems related to scrub typhus: A seriously neglected life-threatening disease. Am. J. Trop. Med. Hyg..

[7] Guo Y., Guo X.G., Song W.Y., Lv Y., Yin P.W., Jin D.C. (2023). Comparison of chiggers (Acari: Trombiculidae, Leeuwenhoekiidae) on two sibling mouse species, *Apodemus draco* and *A. ilex* (Rodentia: Muridae), in Southwest China. Animals.

[8] Dernea B., Weinstein P., Musso D., Lau C. (2015). Distribution of rickettsioses in Oceania: Past patterns and implications for the future. Acta Trop..

[9] Seshkanta L., Eliz A., Satyam M., Rabin G., Amrit A. (2023). A case of acute encephalitis syndrome and cranial nerve palsy secondary to scrub typhus: A rare presentation from Western Nepal. Clin. Case Rep..

[10] Wu Y.C., Qian Q., Soares Magalhaes R.J., Han Z.H., Hu W.B., Haque U., Weppelmann T.A., Wang Y., Liu Y.X., Li X.L. (2016). Spatiotemporal dynamics of scrub typhus transmission in mainland China, 2006–2014. PLoS Negl. Trop. Dis..

[11] Yue Y.J., Ren D.S., Liu X.B., Wang Y.J., Liu Q.Y., Li G.C. (2019). Spatio-temporal patterns of scrub typhus in mainland China, 2006–2017. PLoS Neglected Trop. Dis..

[12] Peng P.Y., Xu L., Wang G.X., He W.Y., Yan T.L., Guo X.G. (2022). Epidemiological characteristics and spatiotemporal patterns of scrub typhus in Yunnan Province from 2006 to 2017. Sci. Rep..

[13] Elders P.N.D., Swe M.M.M., Phyo A.P., McLean A.R.D., Lin H.N., Soe K., Htay W.Y.A., Tanganuchitcharnchaic A., Hla T.K., Tun N.N. (2021). Serological evidence indicates widespread distribution of rickettsioses in Myanmar. Int. J. Infect. Dis..

[14] Win A.M., Nguyen Y.T.H., Kim Y., Ha N.Y., Kang J.G., Kim H., San B., Kyaw O., Htike W.W., Choi D.O. (2020). Genotypic heterogeneity of *orientia tsutsugamushi* in scrub typhus patients and thrombocytopenia syndrome co-infection, Myanmar. Emerg. Infect. Dis..

[15] Brummaier T., Kittitrakul C., Choovichian V., Lawpoolsri S., Namaik-larp C., Wattanagoon Y. (2017). Clinical manifestations and treatment outcomes of scrub typhus in a rural health care facility on the Thailand-Myanmar border. J. Infect. Dev. Ctries..

[16] Chen L. (2022). Study on the path of exchange and cooperation in ecological and environmental protection in China-Myanmar border: Taking Dehong prefecture of Yunnan Province as an example. China-Arab. States Sci. Technol. Forum.

[17] Tian J., Guo X.F., Zhou H.N., Liu Y.H., Yin X.X., Wu C., Yang M.D., Yang Z.H., Li P., Zheng Y.T. (2021). Molecular epidemiological survey of mosquito-borne viral diseases in patients with a fever of unknown origin at the border between China and Myanmar. J. Pathog. Biol..

[18] Deng X.F., Du S.S., Huang X.X., Wang Q., Li A.Q., Li C., Sun L.N., Wu W., Li H., Liu T.Z. (2023). Epidemiological characteristics of hemorrhagic fever of renal syndrome in China, 2004−2021. Dis. Surveill..

[19] Gong S.J., Yang J.H., Zhang Z.C. (2022). Analysis on growth performance of *Betula alnoides* at different altitude in Dehong prefecture. J. Green Sci. Technol..

[20] Ding F., Jiang W.L., Guo X.G., Fan R., Zhao C.F., Zhang Z.W., Mao K.Y., Xiang R. (2021). Infestation and related ecology of chigger mites on the Asian house rat (*Rattus tanezumi*) in Yunnan Province, southwest China. Korean J. Parasitol..

[21] Guo Y., Guo X.G., Peng P.Y., Lv Y., Xiang R., Song W.Y., Huang X.B. (2023). Infestation and distribution of chiggers on the Anderson’s white-bellied rats in southwest China. Vet. Med. Sci..

[22] Peng P.Y., Guo X.G., Jin D.C., Dong W.G., Qian T.J., Qin F., Yang Z.H. (2017). Species abundance distribution and ecological niches of chigger mites on small mammals in Yunnan Province, southwest China. Biologia.

[23] Chen Y.L., Guo X.G., Ding F., Lv Y., Yin P.W., Song W.Y., Zhao C.F., Zhang Z.W., Fan R., Peng P.Y. (2023). Infestation of oriental house rat (*Rattus tanezumi*) with chigger mites varies along environmental gradients across five provincial regions of southwest China. Int. J. Environ. Res. Public Health.

[24] Zhou J.X., Guo X.G., Song W.Y., Zhao C.F., Zhang Z.W., Fan R., Chen T., Lv Y., Yin P.W., Jin D.C. (2022). Preliminary study on species diversity and community characteristics of gamasid mites on small mammals in Three Parallel Rivers Area of China. Animals.

[25] Chen Y.L., Guo X.G., Ren T.G., Zhang L., Fan R., Zhao C.F., Zhang Z.W., Mao K.Y., Huang X.B., Qian T.J. (2022). Infestation and distribution of chigger mites on Chevrieri’s field mouse (*Apodemus chevrieri*) in southwest China. Int. J. Parasitol. Parasites Wildl..

[26] Guo X.G., Dong W.G., Men X.Y., Qian T.J., Wu D., Ren T.G., Qin F., Song W.Y., Yang Z.H., Fletcher Q.E. (2016). Species abundance distribution of ectoparasites on Norway rats (*Rattus norvegicus*) from a localized area in southwest China. J. Arthropod-Borne Dis..

[27] Liu Z., Guo X.G., Fan R., Zhao C.F., Mao K.Y., Zhang Z.W., Zhao Y. (2020). Ecological analysis of gamasid mites on the body surface of Norway rats (*Rattus norvegicus*) in Yunnan Province, Southwest China. Biologia.

[28] Peng P.Y., Guo X.G., Ren T.G., Dong W.G., Song W.Y. (2016). An updated distribution and hosts: Trombiculid mites (Acari: Trombidiformes) associated with small mammals in Yunnan Province, southwest China. Parasitol. Res..

[29] Wei T., Simko V. “Corrplot”: Visualization of a Correlation Matrix 0.92. [R Package]. https://cran.r-project.org/web/packages/corrplot/corrplot.pdf.

[30] Wei X.Y., Liu X.J., Cheng L., Sun L., Pan Y.Y., Zong W.W. (2017). Evaluating medical convenience in ethnic minority areas of Southwest China via road network vulnerability: A case study for Dehong autonomous prefecture. Int. J. Equity Health.

[31] Zhou J.H., Zhang Y.Z., Zhang Y.Z., Yang W.H., Feng Y. (2021). Epidemiological characteristics of hemorrhagic fever with renal syndrome in Yunnan Province, China, 2012–2020. Chin. J. Vector Biol. Control..

[32] Cao R. (2022). Regional tourism economic impact evaluation based on dynamic input-output model. J. Math..

[33] Oglezneva E., Petrova T., Ying J. (2016). Features of language communication in a multicultural community: Russian texts of advertising signboards in the border cities of China. Proc.-Soc. Behav. Sci..

[34] Yang Z.Q., Liu Y.R. (2003). A preliminary list of chigger mites in Hubei Province. Acta Arachnol. Sin..

[35] Xue J., Zhou G.Z., Liu Y.X. (2004). The faunal study of chigger mites in Shandong Province. Chin. J. Vector Biol. Control..

[36] Liu Z.J., Tian Y., Zhou L., Luo F. (2011). List of Vector Species in Northwest China.

[37] Yin J.X., Geater A., Chongsuvivatwong V., Dong X.Q., Du C.H., Zhong Y.H., McNeil E. (2008). Predictors for presence and abundance of small mammals in households of villages endemic for commensal rodent plague in Yunnan Province, China. BMC Ecol..

[38] Li Q., Li X.Y., Hu W.Q., Song W.Y., He S.W., Wang H.J., Hu Z.C., Li M.C., Onditi K.O., Chen Z.Z. (2024). The mammals of Gaoligong Mountain in China: Diversity, distribution, and conservation. Zool. Res. Divers. Conserv..

[39] Li F., Huang X.Y., Zhang X.C., Zhao X.X., Yang J.H., Chan B.P.L. (2019). Mammals of Tengchong section of Gaoligongshan national nature reserve in Yunnan Province, China. J. Threat. Taxa.

[40] Sadanandane C., Elango A., Panneer D., Mary K.A., Kumar N.P., Paily K.P., Mishra B.B., Sankari T., Jambulingam P. (2021). Seasonal abundance of *Leptotrombidium deliense*, the vector of scrub typhus, in areas reporting acute encephalitis syndrome in Gorakhpur district, Uttar Pradesh, India. Exp. Appl. Acarol..

[41] Lv Y., Guo X.G., Jin D.C., Song W.Y., Fan R., Zhao C.F., Zhang Z.W., Mao K.Y., Peng P.Y., Lin H. (2019). Host selection and seasonal fluctuation of *Leptotrombidium deliense* (Walch, 1922)(Trombidiformes: Trombiculidae) at a localized area of southern Yunnan, China. Syst. Appl. Acarol..

[42] Liu Q.Y., Fan R., Song W.Y., Peng P.Y., Zhao Y.F., Jin D.C., Guo X.G. (2024). The Distribution and Host-Association of the Vector Chigger Species *Leptotrombidium imphalum* in Southwest China. Insects.

[43] Yin P.W., Guo X.G., Jin D.C., Song W.Y., Zhang L., Zhao C.F., Fan R., Zhang Z.W., Mao K.Y. (2021). Infestation and seasonal fluctuation of gamasid mites (Parasitiformes: Gamasida) on Indochinese Forest Rat, *Rattus andamanensis* (Rodentia: Muridae) in southern yunnan of China. Biology.

[44] Tilak R., Kunwar R., Wankhade U.B., Tilak V.W. (2011). Emergence of *Schoengastiella ligula* as the vector of scrub typhus outbreak in Darjeeling: Has *Leptotrombidium deliense* been replaced?. Indian J. Public Health.

[45] Pang R., Xu H., Zhang M., Qian F. (2023). Spatial correlation and impact mechanism analysis of cultivated land fragmentation and quality in the Central Plain of Liaoning Province, Northeast China. Land Degrad. Dev..

[46] Zhang L.Y., Wang Z.E.S., Du G., Chen Z.S. (2022). Analysis of climatic basis for the change of cultivated land area in Sanjiang Plain of China. Front. Earth Sci..

[47] Xiang R., Guo X.G., Zhao C.F., Fan R., Mao K.Y., Zhang Z.W., Huang X.B. (2023). Infestation and distribution of gamasid mites on Himalayan field rat (*Rattus nitidus*) in Yunnan Province of Southwest China. Biologia.

[48] Teitelbaum C.S., Amoroso C.R., Huang S., Davies T.J., Rushmore J., Drake J.M., Stephens P.R., Byers J.E., Majewska A.A., Nunn C.L. (2020). A comparison of diversity estimators applied to a database of host–parasite associations. Ecography.

[49] Walther B.A., Morand S. (1998). Comparative performance of species richness estimation methods. Parasitology.

